# Inflammation and endothelial function‐related gene polymorphisms are associated with carotid atherosclerosis—A study of community population in Southwest China

**DOI:** 10.1002/brb3.3045

**Published:** 2023-05-03

**Authors:** Jing Lu, Wei Peng, Xingyang Yi, Daofeng Fan, Jie Li, Chun Wang, Hua Luo, Ming Yu

**Affiliations:** ^1^ Department of Neurology, Geriatric Diseases Institute of Chengdu/Cancer Prevention and Treatment Institute of Chengdu, Chengdu Fifth People's Hospital (The Second Clinical Medical College Affiliated Fifth People's Hospital of Chengdu University of Traditional Chinese Medicine) Chengdu China; ^2^ Department of Gastrointestinal Surgery, Geriatric Diseases Institute of Chengdu/Cancer Prevention and Treatment Institute of Chengdu Chengdu Fifth People's Hospital (The Second Clinical Medical College Affiliated Fifth People's Hospital of Chengdu University of Traditional Chinese Medicine) Chengdu China; ^3^ Department of Neurology the People's Hospital of Deyang City Deyang Sichuan China; ^4^ Department of Neurology Longyan First Hospital Affiliated to Fujian Medical University Fujian China; ^5^ Department of Neurology the Affiliated Hospital of Southwest Medical University Luzhou Sichuan China; ^6^ Department of Neurology the Suining Central Hospital Suining Sichuan China

**Keywords:** carotid atherosclerosis, endothelial function, genetic polymorphism, high‐risk stroke population, inflammation

## Abstract

**Objectives:**

To investigate the relationships between 18 single nucleotide polymorphisms with carotid atherosclerosis and whether interactions among these genes were associated with an increased risk of carotid atherosclerosis.

**Methods:**

Face‐to‐face surveys were conducted with individuals aged 40 or older in eight communities. A total of 2377 individuals were included in the study. Ultrasound was used to detect carotid atherosclerosis in the included population. 18 loci of 10 genes associated with inflammation and endothelial function were detected. Gene‐gene interactions were analyzed using generalized multifactor dimensionality reduction (GMDR).

**Results:**

Among the 2377 subjects, 445 (18.7%) subjects had increased intima‐media thickness in the common carotid artery (CCA‐IMT), and 398 (16.7%) subjects were detected with vulnerable plaque. In addition, NOS2A rs2297518 polymorphism was associated with increased CCA‐IMT, IL1A rs1609682, and HABP2 rs7923349 polymorphisms were associated with vulnerable plaque. Besides, GMDR analysis showed significant gene‐gene interactions among TNFSF4 rs1234313, IL1A rs1609682, TLR4 rs1927911, ITGA2 rs1991013, NOS2A rs2297518, IL6R rs4845625, ITGA2 rs4865756, HABP2 rs7923349, NOS2A rs8081248, HABP2 rs932650.

**Conclusion:**

The prevalences of increased CCA‐IMT and vulnerable plaque were high in Southwestern China's high‐risk stroke population. Furthermore, inflammation and endothelial function‐related gene polymorphisms were associated with carotid atherosclerosis.

## Graphical abstract

Polymorphisms of genes related to inflammation and endothelial function were associated with carotid atherosclerosis. The interactions among ten SNPs strongly synergistically contributed to a higher risk for carotid atherosclerosis.

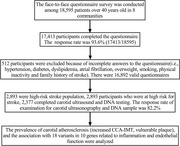

## 1 INTRODUCTION

Stroke is associated with high morbidity and mortality (Liu et et al., al., 2007; Lloyd‐Jones et et al., al., 2009; Mozaffarian et al., 2015). Stroke is caused by a combination of multiple factors, including carotid atherosclerosis, which is strongly associated with ischemic stroke (Ren et al., 2020).

Multiple factors promote the occurrence and development of carotid atherosclerosis, including interactions between traditional risk factors, lifestyle risk factors (age, sex, chronic disease, smoking habit, lack of exercise) and genetic risk factors (Amarenco et al., 2011; Goncalves et al., 2012; Hu et al., 2021; Huang et al., 1999; Lechner et al., 2020; Poznyak et al., 2020). Influence of traditional and lifestyle risk factors, gene‐gene interactions may account for the complexity of carotid atherosclerosis (Yi et al., 2016, 2017). Some studies have shown that some gene loci are closely associated with carotid atherosclerosis (Debette et al., 2006; Humphries & Morgan, 2004). Carotid intima‐media thickening and vulnerable plaque are two types of carotid atherosclerosis (Forgo B et al., 2018). Intima‐media thickness in the common carotid artery is a subclinical marker of atherosclerosis and is associated with an increased risk of stroke (O'Leary et al., 1999). The rupture and detachment of vulnerable plaque are also leading causes of cerebral infarction (Katakami et al., 2010).

Therefore, determining the genetic etiology of carotid intima‐media thickened and vulnerable plaque is critical for preventing atherosclerosis and stroke (Fox et al., 2003; Pitchika et al., 2022).

Technological advancements have allowed for detection and identification of a large number of single nucleotide polymorphisms (SNPs) using matrix‐assisted laser desorption/ionization‐time flight mass spectrometry. Gene‐gene interactions can be analyzed using the generalized multifactor dimensionality reduction (GMDR) approach (Lou et al., 2007). However, few studies have used GMDR to determine genetic factors associated with carotid atherosclerosis.

The risk factors related to carotid atherosclerosis of the high‐risk stroke population in Southwest China were collected. Selected 18 candidate genes involved in inflammation and endothelial function, and investigated the relationships between these SNPs and carotid atherosclerosis (increased CCA‐IMT, vulnerable plaque), GMDR was used to investigate whether interactions among these genes were associated with an increased risk of carotid intima‐media thickening and vulnerable plaque (Lou et al., 2007). Furthermore, analyze whether the collected high‐risk factors would influence the relationship between gene polymorphisms and carotid atherosclerosis.

## 2 MATERIALS AND METHODS

### 2.1 Study design and participants

This multicenter, cross‐sectional survey was a part of the China National Stroke Screening Survey (CNSSS) program of the National Health and Family Planning Commission of China. The survey protocol was reviewed and approved by the Ethics Committee of the participating hospitals (People's Hospital of Deyang City, Suining Central Hospital, and the Affiliated Hospital of Southwest Medical University). Informed consent was obtained from all participants.

**FIGURE 1:**
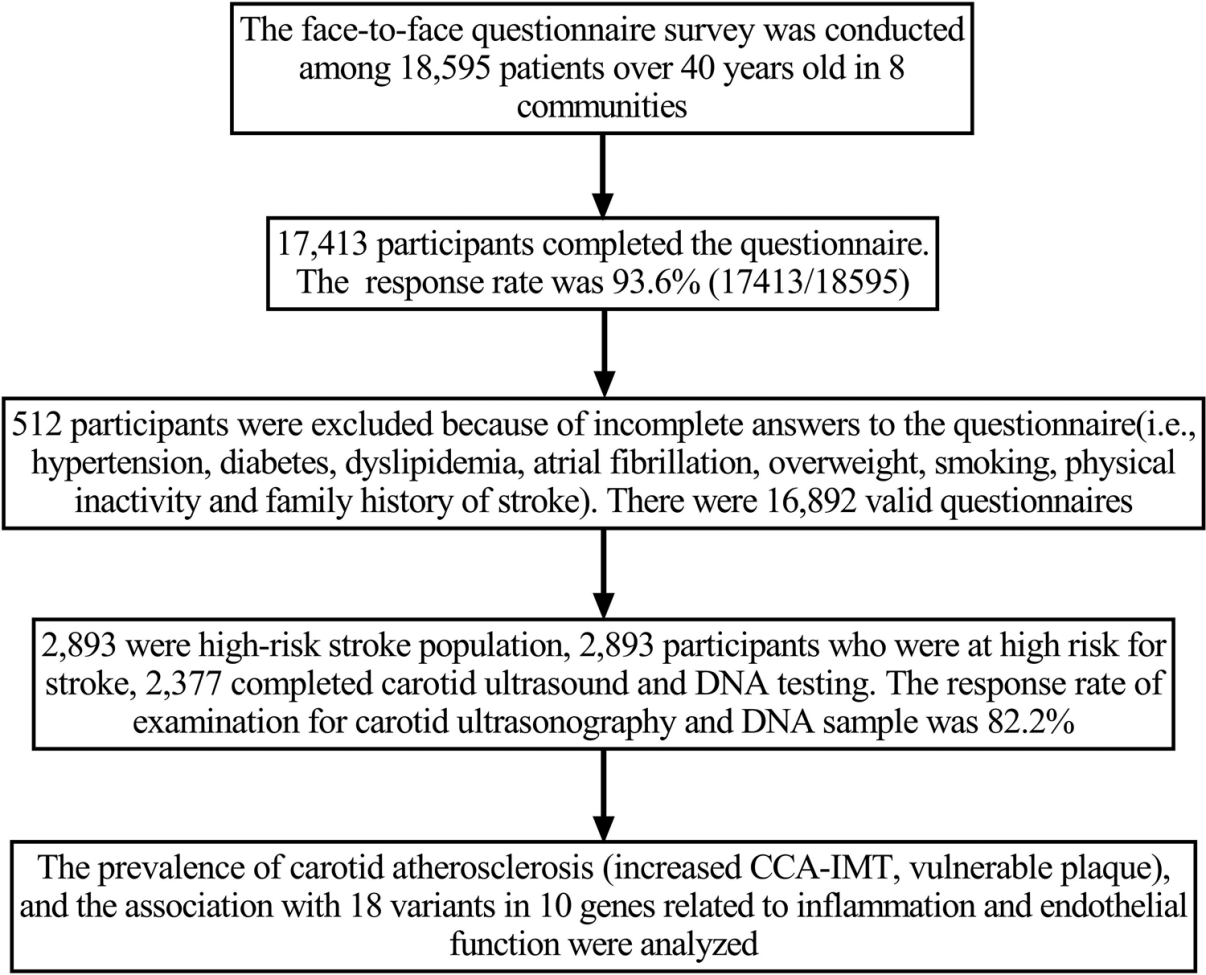
Data preparing and cleaning process in this survey.

Eight communities of Sichuan province in southwestern China were randomly selected, and a cluster survey was performed from May 2015 to September 2015. Details on the study organization, survey, data processing procedures, and quality control were described previously (Yi et al., 2020) and summarized in Figure 1. We initially screened permanent residents aged 40 years or older who had lived in the area for more than 6 months in each community (community residents who lived in Southwest China for a long time had similar eating habits and living habits) using a structured face‐to‐face questionnaire (risk factors of cardiovascular and cerebrovascular diseases in 2015 community screening form) administered by investigators. The questionnaire determined demographic characteristics: age, sex, ethnicity (all respondents were Han), occupation, marital status (unmarried, married, widowed, divorced, other), annual income (< 5000, 5000–10,000, 10,000–20,000, > 20,000), educational level (junior secondary school and below, high school degree or above), behavioral factors: smoking [had smoked continuously or cumulatively for 6 months or more during the lifetime, had smoked in the 30 days prior to the survey, and smoked more than 1 cigarette a day (WHO definition)], drinking [regularly drink large amounts of alcohol (liquor ≥ 3 times per week, ≥ 100 grams each time], lack of physical exercise (physical exercise rarely or light manual workers, less than 30 min each time, less than 3 times a week, lasting ≥ 1 year), personal and family medical history of stroke and chronic diseases: stroke [cerebral hemorrhage, cerebral infarction, and history of stroke were defined as clinical syndromes (WHO definition) of sudden cerebral dysfunction caused by cerebrovascular lesions, lasting for more than 24 h or causing death, and lesions related to post‐stroke symptoms and signs were found on imaging], heart disease (coronary atherosclerotic heart disease, atrial fibrillation, dilated cardiomyopathy, rheumatic heart disease, heart valve insufficiency, heart valve regurgitation), hypertension [systolic blood pressure ≥140 mmHg and/or diastolic blood pressure ≥ 90 mmHg were measured three times on different days without antihypertensive drugs, or systolic blood pressure ≥ 140 mmHg and diastolic blood pressure < 90 mmHg were simple systolic hypertension, or the patient had a history of hypertension and was currently taking antihypertensive drugs, although his blood pressure was lower than 140/90 mmHg, he was also diagnosed as hypertension; Chinese Guidelines for Hypertension Prevention and Treatment (2010 edition)], antiplatelet therapy (aspirin, clopidogrel, ozagrel, dipyridamole/pyridamole, cilostazole, tigrillo), antihypertensive therapy, and physical examination (height, weight, blood pressure). More detailed information on laboratory examinations (such as fasting blood glucose, lipid, electrocardiogram, homocysteine, and carotid ultrasonography) was obtained from individuals who were deemed at high risk for stroke. High risk for stroke was defined as having 1 or more of the following two factors: (1) a history of stroke and (2) three or more of the eight stroke‐related risk factors, including high blood pressure (HP), diabetes mellitus (DM), heart disease, dyslipidemia, overweight or obesity, physical inactivity, family history of stroke, and smoking (Stroke Prevention Project Committee, 2018; Wang et al., 2017). The questionnaire was completed by senior physicians or neurologists in the local community, and they were systematically trained to be familiar with the questionnaire before the survey.

The face‐to‐face questionnaire survey was conducted among 18,595 community residents. A total of 17,413 participants completed the questionnaire (the response rate was 93.6%); 512 participants were excluded because of incomplete answers to the questionnaire (i.e., hypertension, diabetes, heart disease, smoking, and history of stroke). There were 16,892 valid questionnaires. Among 16,892 initially screened participants, 2893 were determined to be high risk for stroke, and 2377 provided DNA data and underwent carotid ultrasound. The remaining high‐risk individuals (516) were excluded because they were unwilling to undergo carotid ultrasound or provide DNA samples due to personal reasons such as life and work, the response rate of examination for carotid ultrasonography and DNA sample was 82.2% (Figure 1).

### 2.2 Carotid ultrasonography measurements

Intima‐media thickness in the common carotid artery and vulnerable plaque were evaluated using high‐resolution B‐mode ultrasound (type 512, Acuson Sequoia Apparatus, 7.5‐MHz probe, Berlin, Germany) (Crouse et al., 1996). The intraobserver and interobserver coefficients were described in our previous study (Yi et al., ^2016^, 2017). Carotid characteristics were evaluated independently by one ultrasound imaging doctor blinded to clinical status. The heads of the patients were turned to the opposite side of the examination before measurement, and vertical scans of the common carotid artery distal wall and the bilateral carotid arteries were taken. The CCA‐IMT was measured at the common carotid artery along the long axis of the blood vessel. Carotid intima‐media thickness was defined as the vertical distance from the tunica intima to the interface between the tunica media and adventitia. The measure was repeated 3 times at this site as well as 1 cm proximal and distal, and the average values were taken as CCA‐IMT. Carotid intima‐media thickening was defined as having an intima‐media thickness ≥ 1.0 mm (Handa et al., 1990; Lorenz et al., 2010; Ma et al., 2016; Spence, 2006). The atherosclerotic plaque was defined as an endoluminal protrusion of at least 1.5 mm or a focal wall thickening >50% than the surrounding vessel wall (Rundek et al., 2008). Based on the plaque echogenicity and surface characteristics, a carotid plaque was further graded from class I to class IV as echolucent, predominantly echolucent, predominantly echogenic, and echogenic, respectively (Mathiesen et al., 2001). Plaque of class I or class II was defined as vulnerable plaque, and plaque of class III or class IV was defined as stable plaque (Yi et al., 2020).

### 2.3 Genes and SNPs selection

Single nucleotide polymorphisms in 10 genes involved in inflammation and endothelial function were selected from the NCBI database (http://www.ncbi.nlm.nih.gov/SNP) according to the following criteria: (1) these SNPs had been evaluated in previous studies (Gardener et al., 2011; Worrall et al., 2003); (2) the eligible SNP had a minor allele frequency > 0.05 (based on the Hapmap plans); (3) the SNPs may result in changes in amino acid sequences. According to these criteria, 18 SNPs from 10 genes implicated in inflammation and endothelial function were evaluated, including PPARA (rs4253778), NOS2A (rs2297518, rs8081248), TNF (rs3093662), IL6R (rs1386821, rs4845625), TNFSF4 (rs1234313, rs11811788), TLR4 (rs752998, rs1927911), IL1A (rs1800587, rs1609682), VCAM1 (rs3783615, rs2392221), ITGA2 (rs1991013, rs4865756), and HABP2 (rs932650, rs7923349).

### 2.4 Primer synthesis

Multiple polymerase chain reaction primers were designed for the qualified SNPs of PPARA (rs4253778), NOS2A (rs2297518, rs8081248), TNF (rs3093662), IL6R (rs1386821, rs4845625), TNFSF4 (rs1234313, rs11811788), TLR4 (rs752998, rs1927911), IL1A (rs1800587, rs1609682), VCAM1 (rs3783615, rs2392221), ITGA2 (rs1991013, rs4865756), and HABP2 (rs932650, rs7923349) using the gene library GenBank (http://www.ncbi.nlm.nih.gov/omim/) and the specificity was identified by ^Primer^BLAST, Sangon Biotech Co, Ltd, Shanghai, China (Table 1).

**TABLE 1:** Amplification and extension primers used in this study.

SNP_ID	forward primer	reverse primer	extension primer
rs4253778	ACGTTGGATGTTCTGGAGATCACAACCACC	ACGTTGGATGCTCCTTAAATATGGTGGAAC	GGAAATGAAGCTTTTGAATC
rs2297518	ACGTTGGATGTTGAGAACTCTGTCATTCCC	ACGTTGGATGAGTAGGACAACGGAAAAAGC	TCTTTCTAGAAACTGAAGAAAT
rs8081248	ACGTTGGATGTTCATCTTCACTGCCCACTC	ACGTTGGATGTCCACAACACCCAGATCAAC	CCCCTCTCATCAGACCCC
rs3093662	ACGTTGGATGGTCACCCTTAAAGGAGGAAC	ACGTTGGATGTGTTGAATGCCTGGAAGGTG	CGGTTTTCTCTCTCCATTCATC
rs1386821	ACGTTGGATGTGCTGTGTGTTAGGGACTTC	ACGTTGGATGTACCTCGAAGGGTGGTTATG	AGGGACTTCTGTAAACACTCTATTT
rs4845625	ACGTTGGATGTTGTCCTTACAGGTCCGATG	ACGTTGGATGTAGCTCGTAAGTGGTGGAAC	CAAGGATGCAGGTACTACC
rs1234313	ACGTTGGATGTAACACTGGCTCTAGTCAAC	ACGTTGGATGCCATTCTGACTAGAATAGGC	GGCTCTAGTCAACACTATAC
rs11811788	ACGTTGGATGTAGGAAGAGGAGATTGAGCG	ACGTTGGATGACCATGGCACCAGATTTCTC	GTATCCTACCATTATGCCA
rs752998	ACGTTGGATGTTGGGACCTGCTCAACTTTG	ACGTTGGATGGAATTCCAGGCAGTCTACAG	ATCTTCATATATGGTTTCCATTTTAAT
rs1927911	ACGTTGGATGCATCACTTTGCTCAAGGGTC	ACGTTGGATGTCAAGATGTCCAGACCTTCC	AAGGGTCAATGAGCCAAG
rs1800587	ACGTTGGATGGGAGAAAGGAAGGCATGGAT	ACGTTGGATGGGCTGGCCACAGGAATTATA	TTTACATATGAGCCTTCAATG
rs1609682	ACGTTGGATGAATGAAAGACAGGTCTCTGG	ACGTTGGATGACACAGTGGTTTCTTCATCC	GTGAGGGTAAAACAAAAGTATT
rs3783615	ACGTTGGATGTATGACCCCTTCATGTTGGC	ACGTTGGATGTTTCTCCTGAGCTTCTCGTG	TGGGCTTTTCTTGCAAAGTAAATTA
rs2392221	ACGTTGGATGTGCACTGGGATGATGCTTAG	ACGTTGGATGGCAGCTTTGTGGATGGATTC	TTATCAATTGCTAATATTATTTTTTGCC
rs1991013	ACGTTGGATGGGAAACGTCTTTTCCTTTGC	ACGTTGGATGTATAGTGGTACAGGAAGTAG	TTTTCCTTTGCACAGAATTATTTA
rs4865756	ACGTTGGATGGGATGAGGTTGCCAGTATTC	ACGTTGGATGGCATGCTCATCCTACCATAC	AGGACTGTGTGCATTCATGCCA
rs932650	ACGTTGGATGGAAGACATTGAGGGTGATAG	ACGTTGGATGATCACAAGGTCGTGGCTAAG	GGGGAGGGTGATAGCTCTCA
rs7923349	ACGTTGGATGGGCTAATTTTACCCCTAGAG	ACGTTGGATGGTACATATACCTAAGGCCCC	CCCCTAGAGAAAAGCTG

### 2.5 SNP detection

Blood samples (3 mL, elbow vein) from the 2377 participants were drawn into sterile tubes containing ethylene diamine tetraacetic acid and were stored at −80°C. DNA extraction and 18 SNPs detection were completed by Sangon Biotech Co, Ltd, Shanghai, China, and purified using the UNIQ‐10 Kit (Sangon Biotech Co., Ltd. Shanghai, China). Genotyping of the 18 SNPs was performed by investigators blinded to the clinical status of the participants using matrix‐assisted laser desorption/ionization‐time of flight mass spectrometry (MassARRAY Analyzer software by Sangon Biotech Co, Ltd, Shanghai, China), as previously described (Yi et al., 2016, 2017).

### 2.6 Statistical analysis

Statistical analysis was performed using SPSS 25.0 (SPSS Inc New York, New York, USA). The results were expressed as percentages for categorical variables, and continuous variables were expressed as the mean ± standard deviation. Baseline clinical characteristics and genotype distributions of the 18 variants were compared using χ^2^ test (categorical variables) and Student's *t*‐test (continuous variables) between subjects with and without carotid atherosclerosis (increased CCA‐IMT, vulnerable plaque).

The allele frequencies for Hardy‐Weinberg equilibrium were assessed using χ^2^ test. Generalized multifactor dimensionality reduction software (β version 0.7, www.healthsystem.virginia.edu/internet/addiction‐genomics/Software) was used to assess gene‐gene interactions among the 18 variants as previously reported (Lou et al., 2007; Yi et al., 2016). Variables that were statistically significant (*p* < .05) in the univariate analysis were subjected to multivariate logistic regression analysis. All tests were two‐sided, and *p* < .05 was considered statistically significant.

## 3 RESULTS

### 3.1 Prevalence of carotid atherosclerosis (increased CCA‐IMT, vulnerable plaque)

The clinical characteristics of the 2377 participants were summarized in Table 2. Among 2377 participants in the high stroke risk population, increased CCA‐IMT was observed in 445 (18.7%) participants and the vulnerable plaque was observed in 398 (16.7%) participants. In the univariate analysis, increased CCA‐IMT was associated with increased age, total cholesterol, low‐density lipoprotein (LDL), decreased high‐density lipoprotein (HDL) levels and being male, history of stroke, antiplatelet, antihypertensive therapy, current smoking, lower educational background. Furthermore, the vulnerable plaque was associated with increased age, total cholesterol and being male, history of stroke, history of hypertension, antihypertensive therapy, current smoking, and regular alcohol consumption, lower educational background. In addition, we found that people without increased CCA‐IMT and vulnerable plaque was more lacking in physical exercise and higher BMI, considering that since we included people over 40 years old, the elderly may rarely reach the amount of exercise required in the questionnaire and for metabolic reasons, they have a higher BMI.

**TABLE 2:** Demographic characteristics of the study population.

	Increased CCA‐IMT (*n* = 445)	Nonincreased CCA‐IMT and vulnerable plaque (*n* = 1695)	*p* Value	Vulnerable plaque (*n* = 398)	Nonincreased CCA‐IMT and vulnerable plaque (*n* = 1695)	*p* Value
Age (years)	64.36 ± 9.56	62.58 ± 9.95	<.01	66.35 ± 8.54	62.58 ± 9.95	<.01
Male (n, %)	229 (51.46)	710 (41.89)	<.01	214 (53.77)	710 (41.89)	<.01
Fasting blood glucose (mmol/L)	6.17 ± 2.37	6.38 ± 2.48	.105	6.61 ± 2.81	6.38 ± 2.48	.109
Triglycerides (mmol/L)	2.00 ± 2.26	1.88 ± 3.88	.539	2.07 ± 2.38	1.88 ± 3.88	.348
Total cholesterol (mmol/L)	5.34 ± 1.07	5.19 ± 1.00	<.01	5.37 ± 1.05	5.19 ± 1.00	<.01
LDL (mmol/L)	3.30 ± 0.96	2.98 ± 0.98	<.01	3.08 ± 0.89	2.98 ± 0.98	.067
HDL (mmol/L)	1.42 ± 0.53	1.62 ± 0.56	<.01	1.61 ± 0.84	1.62 ± 0.56	.834
Homocysteine (mmol/L)	14.16 ± 8.24	13.30 ± 9.11	.070	13.31 ± 6.58	13.30 ± 9.11	.973
Diabetes Mellitus (n, %)	125 (28.09)	458 (27.02)	.652	115 (28.89)	458 (27.02)	.451
Hypertension (*n*, %)	338 (75.96)	1266 (74.69)	.584	317 (79.65)	1266 (74.69)	.038
History of stroke (*n*, %)	108 (24.26)	286 (16.87)	<.01	124 (31.16)	286 (16.87)	<.01
Antiplatelet therapy (*n*, %)	94 (21.12)	276 (16.28)	.016	75 (18.84)	276 (16.28)	.218
Antihypertensive therapy (*n*, %)	212 (47.64)	633 (37.35)	<.01	191 (47.99)	633 (37.35)	<.01
Heart disease (*n*, %)	37 (8.31)	138 (8.14)	.906	30 (7.54)	138 (8.14)	.690
Current smoking (*n*, %)	174 (39.10)	536 (31.62)	<.01	174 (43.72)	536 (31.62)	<.01
Regular alcohol consumption (*n*, %)	121 (27.19)	516 (30.44)	.182	148 (37.19)	516 (30.44)	<.01
BMI (kg/m^2^)	26.11 ± 3.32	26.22 ± 4.08	.561	25.72 ± 3.41	26.22 ± 4.08	.013
Lack of physical exercise (*n*, %)	228 (51.24)	1115 (65.78)	<.01	259 (65.08)	1115 (65.78)	.789
Education level (junior secondary school and below, *n*, %)	401 (90.11)	1465 (86.43)	.039	379 (95.23)	1465 (86.43)	<.01

LDL, low‐density lipoprotein; HDL, high‐density lipoprotein.

### 3.2 Genotype distributions in subjects with and without carotid atherosclerosis (increased CCA‐IMT, vulnerable plaque)

The genotype distributions of the 18 SNPs assessed in this study were consistent with the Hardy‐Weinberg equilibrium (all *p* > .05). NOS2A rs2297518 SNP was significantly associated with increased CCA‐IMT in the single SNPs analysis (Table 3), IL1A rs1609682, and HABP2 rs7923349 SNPs were associated with vulnerable plaque (Table 4).

**TABLE 3:** Genotype distribution in individuals with and without intima‐media thickening (%).

	Increased CCA‐IMT (*n* = 445)	Nonincreased CCA‐IMT and vulnerable plaque (*n* = 1695)	χ^2^ value	*p* Value
TNFSF4 (rs11811788)			1.27	.54
CG	72 (16.18)	260 (15.34)		
GG	6 (1.35)	14 (0.83)		
CC	367 (82.47)	1421 (83.83)		
TNFSF4 (rs1234313)			4.65	.09
AG	210 (47.19)	744 (43.89)		
GG	60 (13.48)	192 (11.33)		
AA	175 (39.32)	759 (44.78)		
IL6R (rs1386821)			0.31	.85
GT	39 (8.76)	136 (8.02)		
GG	1 (0.22)	5 (0.29)		
TT	405 (91.01)	1554 (91.68)		
IL1A (rs1609682)			4.77	.09
GT	212 (47.64)	850 (50.15)		
GG	191 (42.92)	735 (43.36)		
TT	42 (9.43)	110 (6.49)		
IL1A (rs1800587)			0.45	.82
AG	60 (13.48)	219 (12.92)		
GG	383 (86.06)	1464 (86.37)		
AA	2 (0.44)	12 (0.71)		
TLR4 (rs1927911)			1.03	.59
AG	223 (50.11)	810 (47.79)		
GG	153 (34.38)	626 (36.93)		
AA	69 (15.50)	259 (15.28)		
ITGA2 (rs1991013)			0.47	.79
AG	189 (42.47)	740 (43.66)		
GG	42 (9.43)	170 (10.03)		
AA	214 (48.08)	785 (46.31)		
NOS2A (rs2297518)			8.18	.01
AG	98 (22.02)	480 (28.32)		
GG	340 (76.40)	1178 (69.50)		
AA	7 (1.57)	37 (2.18)		
VCAM1(rs2392221)			3.26	.20
CT	115 (25.84)	370 (22.83)		
TT	10 (2.24)	38 (2.24)		
CC	320 (71.91)	1287 (75.93)		
TNF (rs3093662)			0.20	.70
AG	19 (4.26)	81 (4.78)		
AA	426 (95.73)	1614 (95.22)		
VCAM1 (rs3783615)			–	–
AA	445	1932		
PPARA (rs4253778)			1.31	.37
CG	0 (0.00)	5 (0.29)		
GG	445 (100.00)	1690 (99.71)		
IL6R (rs4845625)			0.19	.90
CT	217 (48.76)	840 (49.56)		
TT	122 (27.41)	468 (27.61)		
CC	106 (23.82)	387 (22.83)		
ITGA2 (rs4865756)			3.42	.18
AG	180 (40.44)	624 (36.81)		
GG	232 (52.13)	965 (56.93)		
AA	33 (7.41)	106 (6.25)		
TLR4 (rs752998)			0.63	.73
GT	115 (25.84)	468 (27.61)		
GG	320 (71.91)	1186 (69.97)		
TT	10 (2.24)	41 (2.42)		
HABP2 (rs7923349)			0.19	.90
GT	160 (35.95)	625 (36.87)		
GG	264 (59.32)	996 (58.76)		
TT	21 (4.71)	74 (4.37)		
NOS2A (rs8081248)			0.16	.93
AG	201 (45.16)	756 (44.60)		
GG	194 (43.59)	756 (44.60)		
AA	50 (11.23)	183 (10.80)		
HABP2 (rs932650)			0.04	.98
CT	191 (42.92)	731 (43.13)		
TT	209 (46.96)	798 (47.08)		
CC	45 (10.11)	166 (9.79)		

**TABLE 4:** Genotype distribution in individuals with and without vulnerable plaque (%).

	Vulnerable plaque (*n* = 398)	Nonincreased CCA‐IMT and vulnerable plaque (*n* = 1695)	χ^2^ value	*p* Value
TNFSF4 (rs11811788)			4.45	.10
CG	63 (15.83)	260 (15.34)		
GG	8 (2.01)	14 (0.83)		
CC	327 (82.16)	1421 (83.83)		
TNFSF4 (rs1234313)			0.79	.67
AG	180 (45.23)	744 (43.89)		
GG	49 (12.31)	192 (11.33)		
AA	169 (42.46)	759 (44.78)		
IL6R (rs1386821)			2.69	.25
GT	23 (5.78)	136 (8.02)		
GG	2 (0.50)	5 (0.29)		
TT	373 (93.72)	1554 (91.68)		
IL1A (rs1609682)			9.39	<.01
GT	178 (44.72)	850 (50.15)		
GG	178 (44.72)	735 (43.36)		
TT	42 (10.55)	110 (6.49)		
IL1A (rs1800587)			1.46	.48
AG	60 (15.08)	219 (12.92)		
GG	336 (84.42)	1464 (86.37)		
AA	2 (0.450	12 (0.71)		
TLR4 (rs1927911)			1.31	.51
AG	201 (50.50)	810 (47.79)		
GG	135 (33.92)	626 (36.93)		
AA	62 (15.58)	259 (15.28)		
ITGA2 (rs1991013)			1.26	.54
AG	185 (46.48)	740 (43.66)		
GG	35 (8.79)	170 (10.03)		
AA	178 (44.72)	785 (46.31)		
NOS2A (rs2297518)			0.01	.99
AG	112 (28.14)	480 (28.32)		
GG	277 (69.60)	1178 (69.50)		
AA	9 (2.26)	37 (2.18)		
VCAM1(rs2392221)			1.37	.50
CT	97 (24.37)	370 (22.83)		
TT	10 (2.51)	38 (2.24)		
CC	291 (73.12)	1287 (75.93)		
TNF (rs3093662)			0.18	.69
AG	17 (4.27)	81 (4.78)		
AA	381 (95.73)	1614 (95.22)		
VCAM1 (rs3783615)			–	–
AA	398	1932		
PPARA (rs4253778)			0.02	1.00
CG	1 (0.25)	5 (0.29)		
GG	397 (99.75)	1690 (99.71)		
IL6R (rs4845625)			0.08	.95
CT	199 (50)	840 (49.56)		
TT	107 (26.88)	468 (27.61)		
CC	92 (23.12)	387 (22.83)		
ITGA2 (rs4865756)			0.39	.81
AG	148 (37.19)	624 (36.81)		
GG	222 (55.78)	965 (56.93)		
AA	28 (7.04)	106 (6.25)		
TLR4 (rs752998)			0.56	.75
GT	116 (29.15)	468 (27.61)		
GG	274 (68.84)	1186 (69.97)		
TT	8 (2.01)	41 (2.42)		
HABP2 (rs7923349)			13.28	<.01
GT	159 (39.95)	625 (36.87)		
GG	206 (51.76)	996 (58.76)		
TT	33 (8.29)	74 (4.37)		
NOS2A (rs8081248)			1.09	.57
AG	166 (41.71)	756 (44.60)		
GG	187 (46.98)	756 (44.60)		
AA	45 (11.31)	183 (10.80)		
HABP2 (rs932650)			0.39	.82
CT	172 (43.22)	731 (43.13)		
TT	191 (47.99)	798 (47.08)		
CC	35 (8.79)	166 (9.79)		

### 3.3 Logistic regression analysis of risk factors for carotid atherosclerosis (increased CCA‐IMT, vulnerable plaque)

Logistic regression analysis found that increased CCA‐IMT was associated with increased age (OR = 1.026, 95%CI = 1.014−1.038, *p* < .01), low‐density lipoprotein (LDL) (OR = 1.411, 95%CI = 1.215−1.638, *p* < .01), decreased high‐density lipoprotein (HDL) levels (OR = 0.371, 95%CI = 0.280−0.491, *p* < .01) and being male (OR = 1.455, 95%CI = 1.102−1.920, *p* < .01), history of stroke (OR = 1.771, 95%CI = 1.239−2.531, *p* < .01), antihypertensive therapy (OR = 1.404, 95%CI = 1.116−1.766, *p* < .01) (Table 5). Besides, the vulnerable plaque was associated with increased age (OR = 1.037, 95%CI = 1.024−1.051, *p* < .01), total cholesterol (OR = 1.291, 95%CI = 1.151−1.447, *p* < .01) and being male (OR = 1.765, 95%CI = 1.304−2.388, *p* < .01), history of stroke (OR = 2.416, 95%CI = 1.839−3.173, *p* < .01), antihypertensive therapy (OR = 1.447, 95%CI = 1.107−1.891, *p* < .01), current smoking (OR = 1.382, 95%CI = 1.017−1.879, *p* = .039), lower educational background (OR = 2.744, 95%CI = 1.662−4.530, *p* < .01) (Table 6). Meanwhile, multivariate logistic regression was performed to determine whether NOS2A rs2297518 SNP, IL1A rs1609682, and HABP2 rs7923349 SNPs increased the risk of carotid intima‐media thickening and vulnerable plaque. Variables from the univariate analysis with *p* < .05 were included in the multivariate logistic regression analysis as covariates. After adjusting for covariates, we found that NOS2A rs2297518GG was independently associated with an increased risk of increased CCA‐IMT (OR = 1.362, 95%CI = 1.050–1.766, *p* = .020) (Table 5). Furthermore, IL1A rs1609682TT, HABP2 rs7923349TT were associated with a higher risk for vulnerable plaque (OR = 1.822, 95%CI = 1.201–2.765, *p* = .005 and OR = 1.788, 95%CI = 1.116–2.865, *p* = .016) (Table 6).

**TABLE 5:** GMDR analysis of the best models, prediction accuracies, cross‐validation consistencies, and *p* values for carotid intima‐media thickness.

Best model^a^	Training balanced accuracy	Testing balanced accuracy	Cross‐validation consistency	Sign test (*p* value)
H	0.5351	0.5222	9/10	7 (.1719)
G,Q	0.5528	0.5230	8/10	7 (.1719)
E,F,P	0.5795	0.5297	6/10	8 (.0547)
F,G,M,Q	0.6102	0.5076	4/10	7 (.1719)
B,D,F,G,M	0.6641	0.5143	3/10	7 (.1719)
B,D,G,M,Q,R	0.7371	0.5562	7/10	10 (.0010)
B,D,F,G,M,Q,R	0.8116	0.5642	10/10	9 (.0107)
B,D,F,G,M,N,Q,R	0.8728	0.5589	10/10	9 (.0107)
B,D,F,G,M,N,P,Q,R	0.9116	0.5529	10/10	9 (.0107)
B,D,F,G,H,M,N,P,Q,R	0.9342	0.5719	10/10	9 (.0107)
B,D,F,G,H,M,N,O,P,Q,R	0.9465	0.5672	8/10	7 (.1719)
B,D,F,G,H,I,M,N,O,P,Q,R	0.9553	0.5741	6/10	6 (.3770)
B,C,D,F,G,H,I,M,N,O,P,Q,R	0.9597	0.5624	10/10	5 (.6230)
A,B,C,D,F,G,H,I,M,N,O,P,Q,R	0.9624	0.5709	7/10	5 (.6230)
A,B,C,D,E,F,G,H,I,M,N,O,P,Q,R	0.9634	0.5722	10/10	6 (.3770)
A,B,C,D,E,F,G,H,I,J,M,N,O,P,Q,R	0.9640	0.5769	10/10	10 (.1719)
A,B,C,D,E,F,G,H,I,J,K,M,N,O,P,Q,R	0.9640	0.5769	10/10	6 (.3770)
A,B,C,D,E,F,G,H,I,J,K,L,M,N,O,P,Q,R	0.9640	0.5769	10/10	6 (.3770)

^a^ Numbers A–S represent, rs11811788, rs1234313, rs1386821, rs1609682, rs1800587, rs1927911, rs1991013, rs2297518, rs2392221, rs3093662, rs3783615, rs4253778, rs4845625, rs4865756, rs752998, rs7923349, rs8081248, and rs932650, respectively.

GMDR, generalized multifactor dimensionality reduction.

**TABLE 6:** GMDR analysis of the best models, prediction accuracies, cross‐validation consistencies, and *p* values for vulnerable plaque.

Best model^a^	Training balanced accuracy	Testing balanced accuracy	Cross‐validation consistency	Sign test (*p* value)
P	0.5355	0.5145	8/10	6 (.3770)
G,Q	0.5654	0.5654	10/10	10 (.0010)
E,G,Q	0.5881	0.5280	5/10	8 (.0547)
D,F,M,P	0.6245	0.5166	5/10	6 (.3770)
B,D,F,G,M	0.6848	0.5388	6/10	8 (.0547)
B,D,F,G,M,Q	0.7610	0.5692	9/10	10 (.0010)
B,D,F,G,M,Q,R	0.8371	0.5909	9/10	10 (.0010)
B,D,F,G,M,N,Q,R	0.9014	0.6102	7/10	10 (.0010)
B,D,F,G,M,N,P,Q,R	0.9432	0.6346	10/10	10 (.0010)
B,D,F,G,H,M,N,P,Q,R	0.9636	0.6468	10/10	10 (.0010)
B,D,F,G,H,I,M,N,P,Q,R	0.9740	0.6100	6/10	7 (.1719)
B,D,F,G,H,I,M,N,O,P,Q,R	0.9799	0.6271	7/10	7 (.1719)
B,C,D,F,G,H,I,M,N,O,P,Q,R	0.9832	0.6436	10/10	8 (.0547)
A,B,D,F,G,H,I,M,N,O,P,Q,R	0.9851	0.6526	8/10	8 (.0547)
A,B,C,D,E,F,G,H,I,M,N,O,P,Q,R	0.9859	0.6669	7/10	7 (.1719)
A,B,C,D,E,F,G,H,I,J,M,N,O,P,Q,R	0.9864	0.6676	10/10	7 (.1719)
A,B,C,D,E,F,G,H,I,J,K,M,N,O,P,Q,R	0.9864	0.6676	10/10	7 (.1719)
A,B,C,D,E,F,G,H,I,J,K,L,M,N,O,P,Q,R	0.9864	0.6676	10/10	7 (.1719)

^a^ Numbers A–S represent, rs11811788, rs1234313, rs1386821, rs1609682, rs1800587, rs1927911, rs1991013, rs2297518, rs2392221, rs3093662, rs3783615, rs4253778, rs4845625, rs4865756, rs752998, rs7923349, rs8081248, and rs932650, respectively.

GMDR, generalized multifactor dimensionality reduction.

### 3.4 Gene‐gene interactions among the 18 variants and risk for carotid atherosclerosis (increased CCA‐IMT, vulnerable plaque)

We evaluated the high‐order interaction relationships for the 18 variants with carotid atherosclerosis (increased CCA‐IMT, vulnerable plaque) using GMDR analysis. The results showed significant gene‐gene interactions among genes involved in inflammation and endothelial function. After adjusting for confounding variables, the best interactive model for carotid atherosclerosis (increased CCA‐IMT, vulnerable plaque) was the interaction among TNFSF4 rs1234313, IL1A rs1609682, TLR4 rs1927911, ITGA2 rs1991013, NOS2A rs2297518, IL6R rs4845625, ITGA2 rs4865756, HABP2 rs7923349, NOS2A rs8081248, HABP2 rs932650. The cross‐validation consistency was 10/10—the sign test was 9 (increased CCA‐IMT, *p* = .0107) (Table 7), and the cross‐validation consistency was 10/10—the sign test was 10 (vulnerable plaque, *p* = .0010) (Table 8). This indicated that the interactions among those variants strongly synergistically contributed to a higher risk for carotid atherosclerosis (increased CCA‐IMT, vulnerable plaque) more than any individual variant alone.

**TABLE 7:** Multivariate analysis of the major risk factors for carotid intima‐media thickening.

Risk factor	OR	95% CI	*p* Value
Age	1.026	1.014–1.038	<.01
Male	1.455	1.102–1.920	<.01
Total cholesterol	1.044	0.905–1.205	.555
LDL	1.411	1.215–1.638	<.01
HDL	0.371	0.280–0.491	<.01
History of stroke	1.771	1.239–2.531	<.01
Antiplatelet therapy	0.832	0.572–1.210	.335
Antihypertensive therapy	1.404	1.116–1.766	<.01
Current smoking	1.132	0.853–1.501	.391
lack of physical exercise	0.603	0.482–0.754	<.01
Educational level (junior secondary school and below)	1.205	0.835–1.739	.318
rs2297518			
AG	References		–
AA	0.871	0.360–2.104	.758
GG	1.362	1.050–1.766	.020

OR, odds ratios; CI, confidence interval.

**TABLE 8:** Multivariate analysis of the major risk factors for vulnerable plaque.

Risk factor	OR	95% CI	*p* Value
Age	1.037	1.024–1.051	<.01
Male	1.765	1.304–2.388	<.01
History of stroke	2.416	1.839–3.173	<.01
Hypertension	1.105	0.797–1.532	.549
Antihypertensive therapy	1.447	1.107–1.891	<.01
Current smoking	1.382	1.017–1.879	.039
Regular alcohol consumption	1.141	0.858–1.517	.365
Total cholesterol	1.291	1.151–1.447	<.01
BMI	1.006	0.976–1.038	.680
Educational level (junior secondary school and below)	2.744	1.662–4.530	<.01
rs1609682			
GT	References		
TT	1.822	1.201–2.765	.005
GG	1.094	0.859–1.393	.465
rs7923349			
GT	References		
TT	1.788	1.116–2.865	.016
GG	0.832	0.653–1.059	.135

OR, odds ratios; CI confidence interval.

## 4 DISCUSSION

Traditional nonmodifiable risk factors, lifestyle risk factors, and genetic factors are key drivers of vascular diseases, such as atherosclerosis (Amarenco et al., 2011; Cheng et al., 2016; Goncalves et al., 2012; Hu et al., 2021; Huang et al., 1999; Jiang et al., 2015; Lechner et al., 2020; Poznyak et al., 2020).

Traditional risk factors for atherosclerosis, such as age, sex, history of cardiovascular disease, dyslipidemia, hypertension, and diabetes mellitus are known to predict atherosclerosis risk in the general population (Legge & Hanly, 2018).

With age, the phenomenon of clonal hematopoiesis of indeterminate potential in the bone marrow increases, the function of thread granules decreases, and IL‐6 level in the vasculature increases, all of which promote the formation of atherosclerosis (Tyrrell & Goldstein, 2021).

A study of carotid endarterectomy specimens in men and women showed that the inflammatory infiltration of plaques was more pronounced in men than in age‐matched women, and men had more CD68 staining (an inflammatory marker associated with macrophages) compared with women (Man et al., 2020).

Atherosclerosis is an inflammatory reaction accompanied by vascular endothelial function impairment. In chronic diseases such as hypertension, diabetes, and stroke, the incidences of inflammatory factors and endothelium‐related factors (interleukin‐6, tumor necrosis factor‐α, nitric oxide, reactive oxygen species) increase, metabolic abnormality (small density lipoprotein aggregation, the proinflammatory and immune stimulatory effects of low‐density lipoprotein modified by oxidation or enzymatic modification are mimicked by inflammatory phospholipids, such as lysophosphatidylcholine) leading to the development and aggravation of atherosclerotic lesions, which interact with atherosclerosis (Amarenco et al., 2011; Goncalves et al., 2012; Hu et al., 2021; Huang et al., 1999; Poznyak et al., 2020).

In multivariable analysis, we found that increased age, total cholesterol, low‐density lipoprotein (LDL), decreased high‐density lipoprotein (HDL) levels, and being male, history of stroke were associated with carotid atherosclerosis. This was consistent with previous research findings. However, few previous studies have analyzed the effects of drugs on carotid atherosclerosis; in our study, we included drugs for analysis. Multivariate analysis found that patients with carotid atherosclerosis were more likely to take antihypertensive drugs.

The importance of lifestyle factors beyond traditional risk factors for the prevention of atherosclerosis is receiving increasing attention (Lechner et al., 2020).

We sought to assess the relationship between lifestyle and carotid atherosclerosis in the high‐risk stroke population in Southwest China. In univariate analysis, we found that smoking and alcohol consumption were closely related to carotid atherosclerosis, adjusting for confounding factors in a multifactor analysis found that smoking increased the risk of carotid atherosclerosis. Cigarette smoking is a strong risk factor for the development of certain chronic inflammatory diseases. Smoking can promote the progression of systemic inflammation and promote the occurrence and development of atherosclerosis (Doran et al., 2003; Chang et al., 2014; Papagoras et al., 2014; Gulati et al., 2016; Rodríguez Huerta et al., 2016). Buamina et al. found that there was an interaction between smoking and genes that may promote the development of carotid atherosclerosis (Maitusong et al., 2023). Reducing tobacco inhalation can reduce the incidence of carotid atherosclerosis.

In this study, we found that people without increased CCA‐IMT and vulnerable plaque were more lacking in physical exercise and had higher BMI. But previous research had shown that high leisure‐time physical activity as opposed to low physical activity were associated with reduced risk of incident atherosclerosis (Johansson & Acosta, 2020). We considered that it might be because there were a large number of elderly people in the included population, who lost weight due to diseases, aging, and other reasons that could not reach the exercise tolerance described in the questionnaire. Future age‐stratified assessment investigations are needed to understand the role of these life factors in atherosclerosis.

The interesting finding was that after adjusting for covariates, lower education was associated with a significantly higher risk of vulnerable plaques. To our knowledge, very limited prior studies on the association between education level and atherosclerosis had been conducted in China. One prospective study showed that the incidence of stroke was significantly higher in those with lower education levels than in those with higher education levels, which may be partly attributed to an insufficient understanding of the importance of proper management of cardiovascular risk factors in the population with low education level, as well as the living environment, living habits, and income level were different (Che et al., 2020). Larger prospective studies are needed to analyze the association between education and carotid atherosclerosis.

Genetic factors play an important role in the development of carotid atherosclerosis (Gardener et al., 2011; Grbić et al., 2022). Multiple risk factors may influence the relationship between the gene polymorphisms and carotid atherosclerosis (Li et al., 2022; Maitusong et al., 2023).

Previous studies showed that inflammation and endothelial function‐related functional polymorphisms were associated with ischemic stroke (Cheng et al., 2016; Jiang et al., 2015). However, few studies have focused on carotid atherosclerosis, including carotid IMT and vulnerable plaque. Carotid IMT is an early surrogate marker of subclinical atherosclerosis (Yang et al., 2020) and is an important marker of acute ischemic cerebral infarct (Ravikanth, 2019). Besides, the rupture and detachment of vulnerable plaque are also a leading cause of cerebral infarction (Katakami et al., 2010). Therefore, we investigated the association between variants in genes related to endothelial function and inflammatory processes and possible markers of carotid atherosclerosis (increased CCA‐IMT, vulnerable plaque) in a population of individuals at high risk for stroke in China.

Inflammation induces the proliferation and migration of vascular smooth muscle cells and participates in the regulation of vascular tension to influence the progression and development of atherosclerosis (Gardener et al., 2011; Zhang et al., 2007). Immune cells congregate in intimal neovasculature and inhibit activation of urokinase, resulting in endothelial damage (Galkina & Ley, 2007; Willeit et al., 2003). Genetic polymorphisms associated with inflammation and endothelial function may directly or indirectly interact with vascular risk factors to influence the onset and progression of atherosclerosis (Galkina & Ley, 2007; Gardener et al., 2011; Zhang et al., 2007). Previous studies have shown that multiple risk factors have an impact on the relationship between genes and carotid atherosclerosis, so it is necessary to analyze the correlation between genes and carotid atherosclerosis by combining multiple factors (Li et al., 2022; Maitusong et al., 2023).

Gardener et al. used GoldenGate Assay (Illumina, San Diego, CA) for genotyping. The final set included 694 SNPs. From the 147 candidate genes, 43 genes (197 SNPs) implicated in inflammation and endothelial function were included in their study. And they found nine genes involved in inflammation and endothelial function had SNPs strongly associated (*p* ≤ .01) with any of the carotid plaque phenotypes in the single SNP analysis: TNF, NOS2A, IL6R, TNFSF4, PPARA, IL1A, TLR4, ITGA2, HABP2, and NOS2A (rs2297518, rs8081248) SNPs, IL6R rs1386821 SNP, TNFSF4 rs1234313 SNP, IL1A rs1800587 SNP, ITGA2 rs1991013 SNP, HABP2 rs932650 SNP were significantly associated with carotid plaque (Gardener et al., 2011). But in our single SNPs analysis, we found that only NOS2A rs2297518 SNP was significantly associated with increased CCA‐IMT, IL1A rs1609682, and HABP2 rs7923349 SNPs were associated with vulnerable plaque, no other SNPs were found to be associated with increased CCA‐IMT, vulnerable plaque. In multivariate logistic regression analysis, after adjusting for covariates (included sex, total cholesterol, low‐density lipoprotein, high‐density lipoprotein levels, history of stroke, history of hypertension, antiplatelet therapy, antihypertensive therapy, current smoking, regular alcohol consumption, lower educational background; *p* < .05 variables), we found that NOS2A rs2297518GG was independently associated with an increased risk of increased CCA‐IMT, and IL1A rs1609682TT and HABP2 rs7923349TT were associated with a higher risk for vulnerable plaque.

Multiple genes contribute to increased carotid atherosclerosis (Guo et al., 2020; Wu et al., 2020). Therefore, studying the gene‐gene interactions that contribute to carotid atherosclerosis is essential. In this study, we analyzed the relationships between 18 SNPs and carotid atherosclerosis using GMDR. The results showed significant gene‐gene interactions among TNFSF4 rs1234313, IL1A rs1609682, TLR4 rs1927911, ITGA2 rs1991013, NOS2A rs2297518, IL6R rs4845625, ITGA2 rs4865756, HABP2 rs7923349, NOS2A rs8081248, and HABP2 rs932650. These results suggested that these 10 genes may synergistically promote carotid atherosclerosis (increased CCA‐IMT, vulnerable plaque). Furthermore, these results indicated that carotid atherosclerosis is a complex, multifactorial process.

However, the interactions between the ten genes identified in this study and carotid atherosclerosis have not been characterized. Carotid atherosclerosis is caused by many factors, including endothelial injury, recruitment, and activation of immune/inflammatory cells, an influx of lipoproteins through the vessel injury space, and smooth muscle cell proliferation (Berliner et al., 1995; Hansson, 2001). Numerous studies had evaluated the roles of TNFSF4, IL1A, TLR4, ITGA2, NOS2A, IL6R, and HABP2 variants in inflammation and endothelial function (Gardener et al., 2011; Hayashi et al., 2006; Hulkkonen et al., 2009; Jiang et al., 2019; Matarin et al., 2008; Methe et al., 2005).

TNFSF4 encodes the OX40L protein (Hauser et al., 2004). Jiang et al. found that the A and AA genotypes at the rs1234313 site of TNFSF were significantly associated with atherosclerotic stroke, and TNFSF rs45454293 SNP was associated with carotid plaque thickening in patients with acute ischemic stroke. The TNFSF rs1234313 SNPs increased the incidence of carotid plaque calcification (Jiang et al., 2019).

IL1A plays a vital role in immunomodulatory and inflammatory processes. Meanwhile, it promotes arterial plaque formation by regulating the expression of IL‐1 in plaque macrophages (Kölsch et al., 2007). IL‐1A rs1800587 SNP promotes irregular and carotid plaque formation (Gardener et al., 2011).

TLR4 is a lipopolysaccharide signaling receptor. Activated TLR4 associates with the tumor necrosis factor receptor and can activate NF‐κB, extracellular signal‐regulated kinases, SAPK/JNK, and p38 MAP kinases. Studies have shown that TLR4 promotes carotid atherosclerosis through increased expression of inflammatory cytokines (Methe et al., 2005).

ITGA2 regulates cell adhesion and cell surface‐mediated signaling. Studies have shown that ITGA2 polymorphism was associated with calcified plaques. Furthermore, ITGA2 polymorphism was associated with the risk of ischemic stroke, carotid intima‐media thickening, and plaques in patients with type 2 diabetes (Gardener et al., 2011; Maeno et al., 2002; Matarin et al., 2008).

The NOS2A gene regulates inducible nitric oxide synthase, which produces nitric oxide. Nitric oxide is involved in vascular tone regulation, immune response, and neurotransmission. In humans, inducible nitric oxide synthase has been observed in the cores of carotid plaques, and inhibition of nitric oxide synthase slowed the progression of atherosclerosis in experimental rabbits (Gardener et al., 2011; Hayashi et al., 2006).

IL‐6 is secreted by T lymphocytes and epithelial cells and can promote endothelial cell activation and leukocyte adhesion (Hulkkonen et al., 2009). Studies have shown that IL‐6 promoted increased carotid intima‐media thickness (Rizza et al., 2020). Interleukin‐6 signals through the IL‐6 receptor (IL‐6R). Therefore, the IL6R gene locus may be associated with carotid IMT (Hassan et al., 2020).

Vascular integrity is regulated by HABP2, which encodes a protein involved in cell adhesion. The haploid test showed that HABP2 rs7923349, rs2302373, and rs2240878 SNPs were associated with plaques (Gardener et al., 2011). HABP2 polymorphism is also associated with the progression of carotid artery stenosis (Willeit et al., 2003).

Therefore, these ten genes may promote the onset and progression of atherosclerosis because they encode and regulate enzymes related to inflammation and endothelial function, which are the main pathogenic mechanisms of atherosclerosis. Future studies will use knockout mice to characterize the molecular mechanisms of interaction among the ten gene variants identified in this study.

Our study was subject to the following limitations. First, the study only included people over 40 years and therefore does not represent the general population of Southwest China. Second, this study was a cross‐sectional study and may be subject to recall bias. Third, this study analyzed the associations between gene polymorphism and thickening of the carotid intima‐media, vulnerable plaque in a population at high risk for stroke, but did not simultaneously analyze the association between gene polymorphisms and carotid artery stenosis. Finally, our study only included 18 loci of 10 genes; there may be other gene loci related to carotid atherosclerosis. Future studies should include more genes to illustrate the relationships between genes and carotid atherosclerosis.

## 5 CONCLUSION

Despite the above limitations, the present study provides clear evidence that the prevalences of increased CCA‐IMT and vulnerable plaque were very high in a population of individuals at high risk for stroke in southwestern China. Multivariate logistic regression showed that NOS2A rs2297518GG was associated with carotid intima‐media thickening; IL1A rs1609682TT and HABP2 rs7923349TT were associated with a higher risk for vulnerable plaque. Furthermore, GMDR analysis showed gene‐gene interactions among TNFSF4 rs1234313, IL1A rs1609682, TLR4 rs1927911, ITGA2 rs1991013, NOS2A rs2297518, IL6R rs4845625, ITGA2 rs4865756, HABP2 rs7923349, NOS2A rs8081248, and HABP2 rs932650. Future studies are needed to confirm the significance of our findings.

## CONFLICT OF INTEREST STATEMENT

The authors declare that they have no competing interests.

### PEER REVIEW

The peer review history for this article is available at https://publons.com/publon/10.1002/brb3.3045.

## Data Availability

All data generated during the project will be made available upon the request from the corresponding author. There are no security, licensing, or ethical issues related to these data.
